# Concurrent listening impairs compensatory postural control mechanisms in middle and late adulthood

**DOI:** 10.1371/journal.pone.0321828

**Published:** 2025-04-30

**Authors:** Nathan Van Humbeeck, Mira Van Wilderode, Astrid van Wieringen, Ralf T. Krampe

**Affiliations:** 1 Brain & Cognition Group, University of Leuven (KU Leuven), Leuven, Belgium; 2 Research Group Experimental Oto-Rhino-Laryngology, University of Leuven (KU Leuven), Leuven, Belgium; 3 Department of Special Needs Education, University of Oslo, Oslo, Norway; Polytechnic University of Marche: Universita Politecnica delle Marche, ITALY

## Abstract

Multitasking involving sensorimotor functions has been shown to affect older more than young adults but little is known about whether related challenges already emerge in middle adulthood. Here we compare 21 younger (18–30 years of age), 23 middle-aged (45–65 years of age) and 19 older participants (66–81 years of age) who listened to and memorized spoken words while they tried to maintain a stable posture on a force platform. The number of words in the listening task was adjusted to individual single-task levels and cognitive control demands were manipulated by presenting words from either the same or switching target speakers. Postural control demands were varied by manipulating proprioceptive reliability (stable stance or sway-referencing). Young adults’ listening and postural control remained unaffected by concurrent performances. During multitasking middle-aged and older adults maintained single-task levels in listening, however, their postural stability declined significantly. Stabilogram diffusion analysis identified efficiency and timing of long-term corrective mechanisms as the key processes affected by multitasking. We argue that middle-aged adults can maintain young-adult levels of postural stability under ideal conditions by cognitive compensation for sensorimotor decline, a process breaking down during multitasking. Our findings illustrate that multitasking in ecologically relevant listening and postural control settings presents a significant challenge as early as middle adulthood.

## Introduction

Young adults and normal-hearing older adults typically experience listening as effortless as long as they can focus on the activity and only one speaker is involved. However, even in everyday settings, adverse conditions like background noise or multiple speakers challenge the perceptual, cognitive, and neurophysiological mechanisms underlying listening and speech recognition [1]. Extant models conceptualize listening as ‘hearing with attention and intention’ highlighting its multifaceted nature and the intricate interplay between cognitive and auditory processing [2,3]. In real-life settings like mingling at a reception, communication draws on cognitive control processes to switch attention between speakers, inhibit irrelevant information (e.g., background noise), and update working memory contents to listening goals [4]. Simultaneously we must control our posture to maintain upright stability, orient towards conversation partners and to facilitate non-verbal expression. Although it is widely acknowledged that multitasking involving sensorimotor and cognitive functions poses more challenges for older compared with young adults [5], there is limited consensus on the underlying processes responsible for task interference as well as the specific ages at which performance decrements become evident. Some studies suggested that cognitive-sensorimotor multitasking declines as early as middle adulthood [6–8]. However, the available evidence remains scarce and inconclusive [9].

In the present study, we focus on postural control and listening, two real-life capabilities where middle-aged adults perform at young-adult levels under favorable conditions [10,11], but start to experience age-related declines when challenged [6]. Young, middle-aged and older adults listened to words from male and female speakers with the goal of memorizing its contents while concurrently maintaining a stable stance on a force platform. We manipulated cognitive control demands by having participants either focus on the same target speaker or switch target speakers. Postural control demands were varied by comparing side-by-side stable stance with sway-referencing, a condition affecting proprioceptive reliability where stability must be dynamically adjusted. We used stabilogram-diffusion analysis (SDA) to assess individual differences in the contribution of short- and long-term control mechanisms in postural control. Our goal was to understand how these mechanisms and their interaction are affected by multitasking and at which phase of adulthood these effects become critical. By manipulating the cognitive control demands of the listening task we aimed to disambiguate between cognitive control accounts of multitasking and models postulating the compensatory usage of cognitive processing in adapting to age-related declines in sensorimotor functioning.

Behavioral research on adult aging has traditionally concentrated on later-life declines, emphasizing how alterations in physical, sensory and cognitive domains can lead the way to fall incidents, social isolation, and cognitive impairment. However, these declines are not abrupt, but they develop gradually during middle (or in case of processing speed, young) adulthood. Schaie [12] reported on the lifespan trajectory of intellectual abilities during adulthood using longitudinal data of thousands of individuals followed over several decades. Apart from declines in perceptual processing speed, there was little longitudinal evidence for age decrements in primary cognitive abilities prior to age 60. Various cognitive abilities (i.e., verbal meaning, spatial orientation, inductive reasoning) even showed modest gains when transitioning from young adulthood to early middle age, with peak ages and degrees of age-related changes depending on the ability under consideration. Whereas cognitive abilities remained relatively robust throughout middle adulthood, research uncovered different trajectories for physical and sensory abilities. Longitudinal research indicated a steady decline for muscle strength from age 40 onwards [13]. A similar trend was observed for persons with symmetrical high frequency hearing loss beginning to emerge as early as the fourth decade of life [14]. In studies comparing young and middle-aged individuals under favorable test conditions (i.e., speech in silence, side-by-side stable upright stance) age-differences where small or absent [10,11]. However, studies employing demanding contexts like speech-in-noise perception or postural control during sway-referencing provided clear evidence for the declining motor and sensory abilities during middle adulthood reported in longitudinal studies [15,16].

Dual-task paradigms have been used to determine whether sensorimotor functions compete with cognitive functions for processing resources. The original general resource account departed from the notion that task performance is inherently dependent on a finite pool of domain-general resources [17]. Once task demands exceed the individual’s capacity, performance decrements will arise. In its generic form, the general resource account is as intuitively appealing as it is circular as it does not specify the neurocognitive processes underlying the resource concept [18]. Likewise, the question remained whether resources are just “drained” by competing tasks or whether resource allocation processes must be considered. Several authors indeed argued that domain-general cognitive control processes like task-switching or inhibition play a central role in multitasking and this assumption was supported in imaging studies [19,20]. Cognitive control accounts of multitasking predict that costs of concurrent performance should reflect the degree to which component tasks by themselves draw on cognitive control processes. This was indeed confirmed in several studies. Meijer & Krampe [21] had young and older adults perform simple mental arithmetic tasks along with movement timing tasks. When the cognitive task required switching of mental sets (i.e., meaning of numbers to be added) dual-task costs increased, particularly in the elderly.

Age-related declines in domain-general functions like processing speed or working memory capacity have been considered as markers for reduced resources and critical factors in limiting multitasking abilities in the elderly [22,23]. Based on age-related changes in cognitive control functions like task-switching and inhibition [24,25] cognitive control theories of multitasking arrive at similar predictions. Studies on multitasking comparing young and older participants generally support the assumption of larger multitasking costs for older compared with young adults. This is even true when seemingly automatic sensorimotor functions like walking or postural control are performed concurrently with cognitive tasks [5,26]. Several authors have argued for a heightened coupling of cognitive and sensorimotor abilities with increasing age [27–30]. Age-related alterations in balance [31,32], hearing [33,34] and multisensory processing [35] have been repeatedly linked to cognitive decline [36]. Age-related hearing loss was found to be the largest potentially modifiable risk factor for development of dementia [37], and a combination of slow gait and subjective cognitive complaints can be indicative of a predementia syndrome (i.e., motoric cognitive risk syndrome [38]). An account specifically targeting adult-age differences in cognitive-sensorimotor multitasking is the cognitive compensation hypothesis proposed by Li and Lindenberger [30]. The authors argue that older adults utilize cognitive functions to partially compensate for changes in sensory processing. As a result, processing resources dedicated to cognitive processes in young adults are permanently reserved to support sensorimotor functions in older adults thereby limiting cognitive efficiency. At the same time, adaptive compensation enables aging individuals to maintain near young-adult levels of sensorimotor functioning. A related account emphasizing the specific role of sensorimotor functions in later adulthood was proposed by Heuninckx et al. [39]. These authors argued that in older adults sensorimotor processing is less automatized compared to young adults. Similar to the cognitive compensation account, lack of automaticity increases demands for cognitive processing in sensorimotor-cognitive multitasking resulting in higher costs for older adults.

General resource, cognitive control and cognitive compensation accounts arrive at similar predictions when it comes to increased multitasking costs in later adulthood. This is partly due to the fact that the accounts are not mutually exclusive, but it also reflects our limited insights into underlying neurocognitive mechanisms. Neuroimaging studies of sensorimotor tasks (e.g., postural control, walking) have been constrained by the limitations imposed by certain techniques, such as MRI, which often require participants to remain motionless. While research on upper and lower limb motor control has shown increased neural recruitment with age [39–42], recent advancements in functional near-infrared spectroscopy (fNIRS) offer a promising alternative for studying sensorimotor functions during movement. St. George et al. successfully employed fNIRS to investigate prefrontal cortex (PFC; associated with cognitive control) activity during single and dual-task walking [43]. Results indicated increased PFC activation during walking in older when compared with younger adults. Moreover, interference between walking and the concurrent task was increased when concurrent cognitive tasks themselves involved cognitive control processes. While these studies offer exciting new insights, they cannot fully disambiguate the different accounts of multitasking. Increased involvement of cognitive control processes, cognitive compensation or lack of automatization likewise imply increased prefrontal activity.

A key methodological problem in investigating age-related differences in multitasking is that large performance differences often already exist at single-task levels [44,45]. For example, presenting identical cognitive tasks to young and older adults often overchallenges older and underchallenges young adults. Related floor-or ceiling effects in single-task conditions render the detection of dual-task costs a mission impossible. Along the same lines, underchallenging in single-task conditions leaves considerable reserve capacity for a secondary task, while exploiting all resources at single-task levels results in an overestimation of dual-task costs. Both scenarios also rule out determining the role of resource allocation problems. Several authors have recommended to individually adjust the difficulty levels of the cognitive task in the single-task condition to allow for comparable resource demands at that level [8,46]. This is a crucial prerequisite for a delineation of differential cognitive costs for the concurrent sensorimotor tasks and the role of resource allocation mechanisms (cognitive control).

While resource shortages, cognitive control deficits, and need for compensation seem plausible for elderly individuals, this seems less apparent for healthy middle-aged individuals. In contrast to the large body of literature describing age-related differences in multitasking in older age, few studies have explored cognitive-motor multitasking in middle-aged adults. The available evidence is scarce and ambiguous. Lindenberger et al. [8] found higher dual-task costs with age from middle-adulthood onwards when combining walking and memory encoding. Helfer et al. [6] found higher dual-task costs in middle-aged compared with young adults who concurrently performed postural control and sentence repetition tasks in the presence of steady state noise or speech maskers. In contrast, Naaman et al. [9] showed that while middle-aged adults performed poorer than young adults when walking in unfavorable conditions, the addition of a cognitive subtraction task did not yield age-differential dual-task decrements.

In the present study we combine tasks from domains (listening and postural control) which pose heightened challenges long before late adulthood to determine whether multitasking already declines during middle adulthood. Postural control has enjoyed considerable attention in age-comparative multitasking research. However, most studies used gross summary measures like path length or center-of-pressure (CoP) area. Recently, researchers have criticized these measures for their mathematical limitations and insufficient sensitivity to age-related differences or effects of contextual factors like concurrent task demands [47–51]. While various nonlinear analyses have been proposed to address these limitations (e.g., wavelet transform, multiscale fuzzy entropy analysis, recurrence quantification analysis), one method that has gained considerable attention due to its presumed link to underlying neurophysiological mechanisms is stabilogram-diffusion analysis (SDA) [52–54].

SDA is based on CoP trajectories assessed through a force platform. By averaging squared CoP displacement for multiple time intervals (typically from 1 to 10000 ms) a function described by four parameters can be estimated that depict the efficiency of postural control on short and long timescales. The parameters comprise the transition point separating short and long timescale regions (i.e., critical time & displacement), and the slopes characterizing short- and long-term displacements (diffusion). While short-term diffusion is assumed to mostly reflect biomechanical factors, long-term diffusion is associated with postural corrections based on sensory feedback. The transition between short- and long-term postural-control processes is related to the point in time as well as the displacement when feedback-based corrections start dominating over unsupervised short-term diffusion. Van Humbeeck et al. showed that this critical timepoint and the related displacement can be flexibly adjusted to the individual's capacity as well as the specific task demands [48]. These adjustments as well as optimized long-term corrective processes allowed middle-aged and older adults to partly compensate for increased short-term diffusion.

SDA parameters can be computed separately for anterior-posterior (AP) and medio-lateral (ML) directions, a separation which appears to be critical when considering age differences [45,55–58]. In a more recent study, Van Humbeeck et al. [45] used SDA to investigate age-related differences in concurrent visuospatial working memory and postural control performances in a lifespan sample. Their results pointed to the critical time interval and long-term diffusion as the processes sensitive to multitasking in all groups. The increase in ML long-term diffusion was pronounced in older compared with young adults, pointing to age-differential interference of cognitive processing (working memory) and feedback-based postural corrections. While middle-aged adults showed higher multitasking costs in postural control than young adults did, this may have been the result of their prioritization of working memory performance: middle-aged adults showed the smallest, non-significant reductions in WM performance of all groups. As a result, overall multitasking costs in middle-aged adults were similar to young adults, who had the smallest costs in postural control. The design of the study did not allow to address the question whether age-differential multitasking costs were related to domain-general cognitive control processes.

In the present study, we used a dual-task paradigm that mimicked real-life multitasking challenges for postural control and listening. Our choice of tasks was motivated by our primary goal to determine the onset ages of multitasking limitations when sensorimotor components vulnerable to changes in middle adulthood are involved. By applying SDA we investigated whether the compensatory mechanisms we observed in the earlier study remained effective under dual-task conditions. Following a cognitive compensation account, we hypothesized that challenging postural control demands with a concurrent task relying on functions displaying early age-related deterioration should lead to pronounced multitasking costs from middle adulthood onwards.

Our second goal was to evaluate the explanatory potential of the cognitive control account of multitasking. To this end we used a switching manipulation in the listening task to manipulate cognitive control demands. We expected this manipulation to reduce listening performance and more so for middle-aged and older adults given age-related increases in (global) switching costs [59,60]. From the cognitive control account of multitasking we expected pronounced dual-task costs in the switching speaker condition particularly for the two older groups.

Finally, we manipulated postural control demands through contrasting quiet stance with sway-referencing conditions which was previously shown to provoke age-differential prioritization patterns [61–63], that is, a protective “posture first” strategy in older individuals. Our goal was to determine whether our task combination would elicit such protective behaviors already in middle-aged individuals despite their comparatively small risk of falling.

## Materials and methods

### Participants

A total of 22 younger (18–30 years of age), 24 middle-aged (45–65 years of age) and 23 older adults (66–81 years of age) were recruited through word of mouth, advertisements in local stores, and the KU Leuven’s recruitment software SONA. We did not include participants if they had (1) an acute musculoskeletal disorder affecting balance, (2) a neurological disorder affecting balance (e.g., stroke), (3) a recent history of falls (6 months), (4) self-reported signs of depression (5) consumed alcohol 24h before testing. Participants were screened for cognitive impairment using a modified version of the Cognitive Disorders Examination [64]. One middle-aged and four older participants were excluded based on having a high risk for dementia (score D on the CODEX). Data from one young adult was not included due to technical malfunctioning related to the postural control recordings. Descriptive characteristics of the final sample are presented in Table 1. Written informed consent was obtained from all participants. Participants received a compensation of 8 euros per hour for participation. Participant recruitment and data collection were conducted between July 2021 and May 2024. The study was approved by the medical ethical committee of the KU Leuven (S63310).

**Table 1:** Descriptive characteristics of each age group. Norm-referenced percentiles are based on Hemmelmann et al. (2010) [65].

Age group	Age			Sex				Body mass index	
**Mean**	**SD**	**N**	**Male**	**Female**	**Height**	**Mass**	**Mean**	**Norm-referenced percentile**
Young adults (18–25 yo)	21.81	1.72	21	8	13	172.76	64.50	21.45	30.15
Middle-aged adults (47–65 yo)	56.74	5.15	23	9	14	171.43	75.28	25.64	32.60
Older adults (66–81 yo)	72.58	5.07	19	7	12	166.89	79.75	28.61	54.39

SD, standard deviation; N, sample size; yo, years old

### Procedure

Testing was conducted in two sessions, each lasting approximately 90 minutes. Participants performed listening tasks and postural control tasks, assessed in isolation (single-task context) and concurrently (dual-task context). The first session focused on individually calibrating the listening task difficulty and familiarizing participants with the tasks by performing all experimental combinations twice. During the second session, all experimental combinations were assessed four times. Both sessions were conducted with at least 24 hours and a maximum of 7 days between sessions.

### Listening task

The listening task included an encoding and a recognition phase. The encoding phase required participants to listen to a sequence of words presented binaurally via Peltor H7A headphones while looking at a display. Half of the words were targets which had to be memorized, while the remaining half served as non-targets which had to be inhibited/ignored. Each word was presented by either a male or female speaker, spatially separated by +/- 25°, with the speaker location remaining constant within each trial. At the beginning of each trial, an image (depicting a male or female face) was presented on the screen. This image indicated the target speaker, signaling participants to memorize only the words spoken by that speaker.

**Fig 1:**
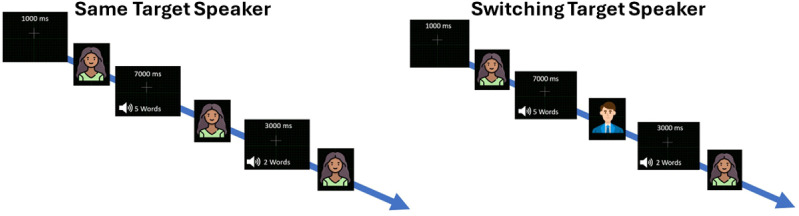
Schematic representation of the listening task in same target speaker and switching target speaker conditions.

The task included two conditions (for an illustration, see Fig 1). In the same speaker condition, the target speaker remained the same throughout the trial, with the starting image occasionally reappearing for a brief duration (300ms) inbetween consecutive words. In the switching target condition, the image that occasionally appeared in between words alternated between the male and female image, requiring participants to switch task sets and update the target speaker based on the most recently presented image. The total duration of the encoding phase was 34 seconds.

During the recognition phase, a new sequence of words was presented visually on the screen. This sequence contained all target words as well as half of the non-target words and a same number of non-presented words. Participants were instructed to press buttons (green or red) based on whether or not the visually presented word matched one of the memorized target words. The goal of the task was to respond as accurate and as fast as possible.

The software APEX [66] was used for auditory presentation, with words being presented at an intensity of 65 dBSPL. If pure tone audiometry indicated abnormal hearing, an individualized filter was used to mitigate differences in audibility by increasing the loudness of the auditory stimuli according to the method used in Van Wilderode et al. [16]. Participants with a hearing threshold exceeding 20 dB HL (decibel hearing level) in at least one frequency (250, 500, 1000, 2000, 4000 kHz) received linear frequency-specific amplification during the listening task. Amplification was applied in 3 young, 11 middle-aged and 15 older adults.

All words presented throughout the experiment are common nouns, with an age of acquisition under 15 (meaning that all selected words are familiar to a typical 15-year-old adolescent) and a concreteness index above 2.5 (indicating that the included words refer to tangible things such as objects and animals rather than abstract concepts) [67].

Each sequence of words presented to the participant was constructed to control for dissimilarities in word prevalence, word length and reaction times based on a lexical decision task [67]. The interstimulus interval between words varied randomly between 1000 ms and 4000 ms. The number of words presented during testing varied based on the preceding individual calibration procedure. Only trials from the second session were included in the data analysis. The percentage correct scores and mean reaction times were provided as feedback after each trial.

When the listening task was performed in isolation (single-task context), the task was performed seated using a 15.6″ Dell laptop screen for visual presentation. When the listening task was combined with the postural control task (dual-task context), participants were standing upright using a 15” Planar PL1500M display for visual presentation. The laptop screen was configured to align with the resolution and aspect ratios of the Planar PL1500M screen in an effort to minimize potential discrepancies.

### Postural control task

For the postural control task, the NeuroCom Balance Master® system was utilized. Participants were instructed to stand as still as possible while maintaining their gaze on a fixation cross, which was displayed at eye level on a screen. Center-of-pressure (CoP) movements were recorded at a sampling frequency of 100 Hz using the integrated 46 cm × 46 cm force plate. Participants stood with their feet positioned approximately shoulder-width apart, oriented forward in a comfortable posture. Participants were required to minimize their bodily movements for 34 seconds.

The task included two conditions. In the stable stance condition, the platform stayed static throughout the trial duration. however, in the sway-referenced condition, the platform rotated around its mediolateral axis based on the real-time position of the participants’ CoP. This means that if the participant is leaning forward, the platform will pitch down, stabilizing movement at the level of the ankle joint. This near-constant angle at the ankle joint will reduce the reliability of the proprioceptive system and presumably increase visual and vestibular contributions. Quantification of the postural control performance was based on the ellipse area as well as stabilogram diffusion analysis (see preprocessing section).

The postural control single-task context comprised a secondary task aimed to make single- and dual-task context equivalent with regard to auditory stimulation. This control task required the participants to listen to a sequence of numbers presented at interstimulus intervals similar to the listening task. Within each trial, all but one (or two) numbers presented were the same (e.g., 7, 7, 7, 7, 7, 5, 7, 7). Participants were asked to remember the last number that was the odd one out. All participants successfully completed the control task without mistakes.

### Individual difficulty calibration

An individual difficulty calibration was performed to ensure that the number of words presented during the listening task posed a sufficient challenge to all participants. The calibration utilized the “same target speaker” condition of the listening task, adaptively adjusting the number of words during two blocks of two trials. During the first block, 12 words were presented in each trial. If the averaged percentage correct score of this block was higher than 85% or lower than 67%, the word length was adjusted by two in order to increase or decrease the difficulty respectively. A similar procedure was applied to the second block. This calibration individualized the task demands (ranging from 10 to 16 words) in an effort to provide a more comparable single-task challenge in the listening task. After this adjustment, the task demands remained fixed within each participant.

### Pure tone audiometry

**Fig 2:**
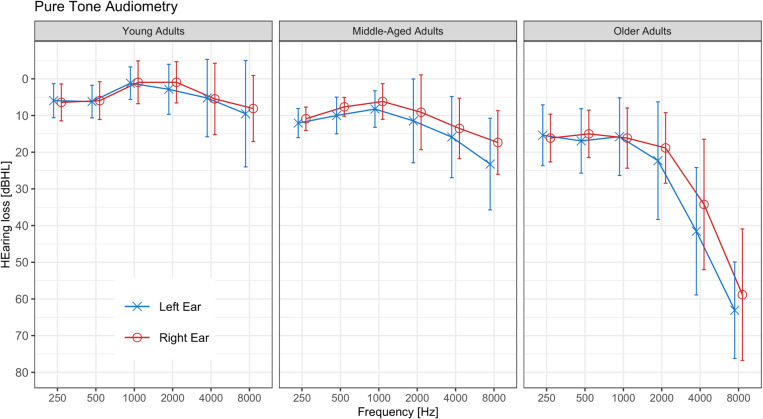
Mean hearing thresholds of the left (blue) and right (red) ear for each age group. Error bars represent standard deviations across participants.

Pure tone audiometry assessed individual hearing thresholds across different frequencies (250, 500, 1000, 2000, 4000 and 8000 kHz). An overview of mean hearing thresholds is depicted in Fig 2.

### Counterbalancing

Experimental conditions included all single-task conditions (postural control stable stance, postural control sway-referenced, listening same target speaker, listening switching target speaker) as well as all combinations of listening and postural control conditions performed concurrently (i.e., stable stance x same target speaker, stable stance x switching target speaker, sway-referenced x same target speaker, sway-referenced x switching target speaker).

Experimental conditions were counterbalanced across participants with two restrictions. The task context manipulation was implemented as an ABBA sequence in which all participants first performed half of the single-task blocks (A) followed by the dual-task blocks with a break in between half of the trials (B-break-B) and the second half of single-task blocks (A). This approach optimally balances training, transfer and fatigue effects for the task-context manipulation. The second restriction was that the order of tasks and task difficulty remained the same across single- and dual-task conditions.

### Preprocessing

This study utilized R version 4.1.2 (R Core Team, 2021) for all preprocessing and statistical analyses. To remove high-frequency noise from the CoP data, a fourth-order Butterworth low-pass filter with a cutoff frequency of 13 Hz was applied [68]. To quantify postural control according to traditional summary statistics, the ellipse area including 95% of the CoP datapoints was included [69]. Additionally, component processes of postural control were assessed using stabilogram diffusion analysis (SDA) [52]. The methodology for calculating SDA parameters aligned with the approach described in Van Humbeeck et al. [48] and involved calculating the mean squared displacement of all data point pairs separated by up to a 10-second window (stabilogram diffusion function). Based on these stabilogram diffusion functions and utilizing the segmented function from the segmented package in R, short and long-term control processes as well as their transition were estimated for each postural control trial.

### Statistical analysis

Data normality checks and transformations were performed prior to statistical analyses. The distributions of the dependent variables were assessed using the Box-Cox method and the check_model function from the car and performance packages in R. These analyses indicated that logarithmic transformations were necessary to achieve normality for all variables except the long-term diffusion coefficient. Since Box-Cox transformations require strictly positive data, they could not be applied to the long-term diffusion coefficient, which included approximately ±7% of values below zero. To accommodate negative values, a modified Box-Cox method (BcnPower) was used. This approach recommended and applied a negative power transformation with lambda (λ) set to 0 and gamma (ɣ) set to 0.2.

We first evaluated the effectiveness of our individualized auditory working memory demands in the listening task. This involved comparing same speaker condition percentage correct scores and number of words presented across age groups. Next, linear mixed-effects models [70] were used to assess the impact of multitasking on both listening and postural control performance. For the listening task, percentage correct scores were analyzed to assess single- and dual-task performances under all conditions. For the postural control task, ellipse area as well as SDA parameters separated by anteroposterior and mediolateral directions served as dependent variables. Finally, z-transformed performance measures were employed to analyze performance across both tasks in a single analysis.

Base models included factors for context (single, dual-easy, dual-difficult), age group (young adults, middle-aged adults, older adults), task condition (easy, difficult), Sex (male, female) and subject as a random factor. Context comparisons were evaluated with 2 prespecified contrasts: one comparing single- with the average of the dual-task conditions, and the other comparing the first with the second dual-task condition. Age group contrasts included a repeated contrast [71], comparing young to middle-aged and middle-aged to older adults. To explore potential developmental trends within age groups, we incorporated exact age (centered) as a nested factor within each age group. When analyzing differences between multitasking costs in different modalities, we z-transformed the performance measures in each of the task modalities and calculated the multitasking costs by subtracting the mean dual-task performance from the mean single-task performance in each experimental condition for each subject. In these models we also included factors for modality (postural control, listening task), primary task (easy, difficult) and secondary task (easy, difficult).

We used backwards exclusion to obtain an optimal parsimonious model fit (i.e., fewest necessary degrees of freedom). First, we assessed whether removing interactions between factors and/or nested effects contributed to the model fit, removing all interactions at p > 0.05. We then evaluated whether expanding the random effect structure by including interactions between subject, context, and/or modality could further improve the model fit.

## Results

### Effectiveness of individual adjustment working memory demands

**Fig 3:**
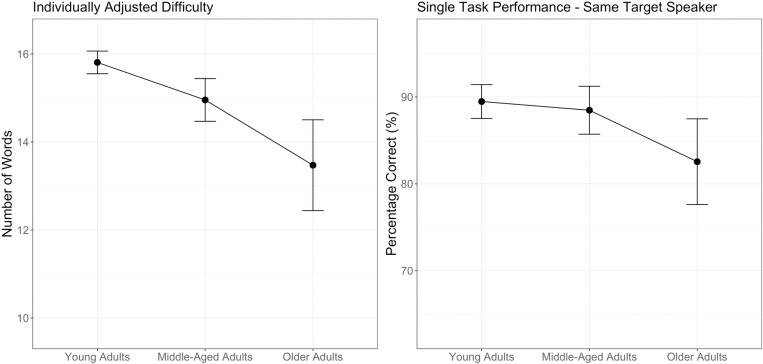
Number of words presented in the listening task (left panel) and associated single-task performance in the same target speaker condition (right panel) between age groups. Error bars represent 2 standard errors.

Fig 3 (left panel) shows the individually adjusted number of words presented during the listening task. A one-way ANOVA using our prespecified age-group contrasts revealed that middle-aged adults performed the task with significantly more words compared with older adults (F(1)=21.821, p<.001). While older adults worked with fewer stimuli in the listening task compared with the other age groups, they nonetheless showed lower percentage correct scores in single-task same target speaker conditions (Fig 3, right panel), as substantiated by a one-way ANOVA indicating robust differences between middle-aged and older adults (F(1)=10.434, p=.002).

### Listening

**Fig 4:**
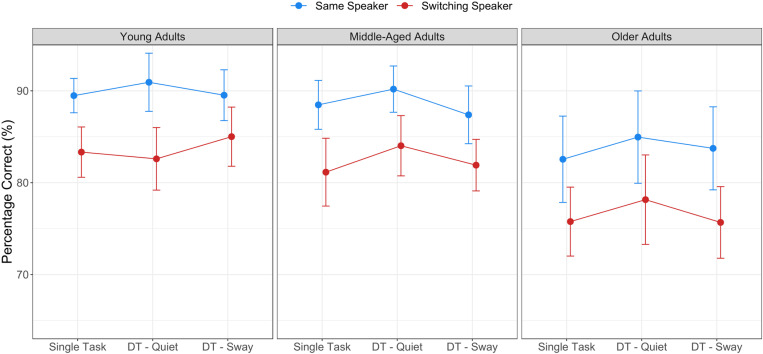
Listening task performance under same and switching target speaker conditions in single- and dual-task contexts (DT – Quiet, DT – Sway). Error bars represent 95% between-group confidence intervals.

Fig 4 illustrates that our switching target speaker manipulation was successful in providing increased challenges (z=10.009, p<.001), and this was the case for all age groups (t’s>4.754, p’s<.001). Different from our expectations, switching effects were similar across age groups. Overall, middle-aged adults performed at similar levels as young adults did, but reliably better than older adults (z=3.421, p=.001). Lastly, nested effects of age revealed that percentage correct scores in middle-aged (z=2.901 p=.005) and older adults (z=3.863, p<.001) decreased with increasing age. For more details on the effects of word category, response times, and task strategies, see S1 Appendix.

### Postural control (summary statistics)

**Fig 5:**
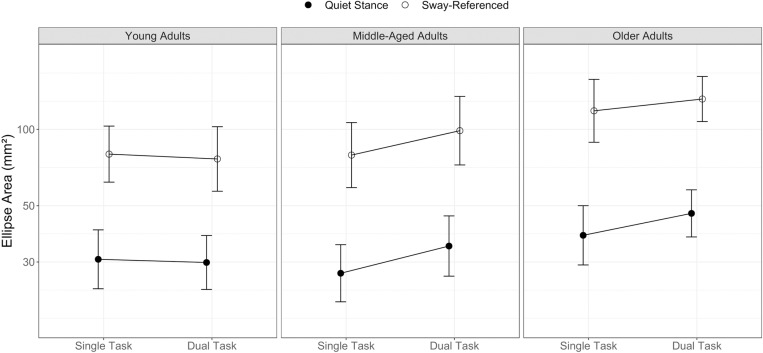
Postural control performance according to the CoP ellipse area under side-by-side quiet and sway-referenced conditions in single and dual task contexts. Error bars represent 95% between-group confidence intervals.

We first analyzed postural control using a common summary statistic, ellipse area (Fig 5). As expected, all groups were less stable (swayed more) in the sway-referenced compared with the quiet stance condition (z=19.504, p<.001). Different from our expectations, we found no indication for effects of listening-task difficulty (same vs. switching speakers; z=0.108 p=.914) and this was the same across age groups and posture-task conditions. We found a main effect of context (z=3.063, p=.003) that was qualified by an interaction with the young vs middle-aged contrast. While middle-aged adults achieved the same stability as young adults in single-task conditions, they showed significantly higher multitasking-related increases in sway compared with younger adults (z=3.449, p=.001).

Post-hoc t-tests averaged across quiet and sway-referenced conditions revealed reliable differences between single- and dual-task contexts in middle-aged adults (t(22)=4.178, p<.001). The difference in older adults was not reliable (t(18)=1.830, p=.084). When only quiet side-by-side stance was considered, both middle-aged (t(22)=3.377, p=.003) and older adults (t(18)=2.134, p=.047) showed reliable dual-task performance decrements. Overall, older adults showed more sway than middle-aged adults (z=2.080, p=.042), while context effects did not differ between the two groups (i.e., no group by context interaction, z=-1.024, p=.310). Male participants showed higher ellipse areas when compared with female participants (z=2.992, p=.004), but this effect was similar across age groups and conditions.

### Postural control (SDA)

**Fig 6:**
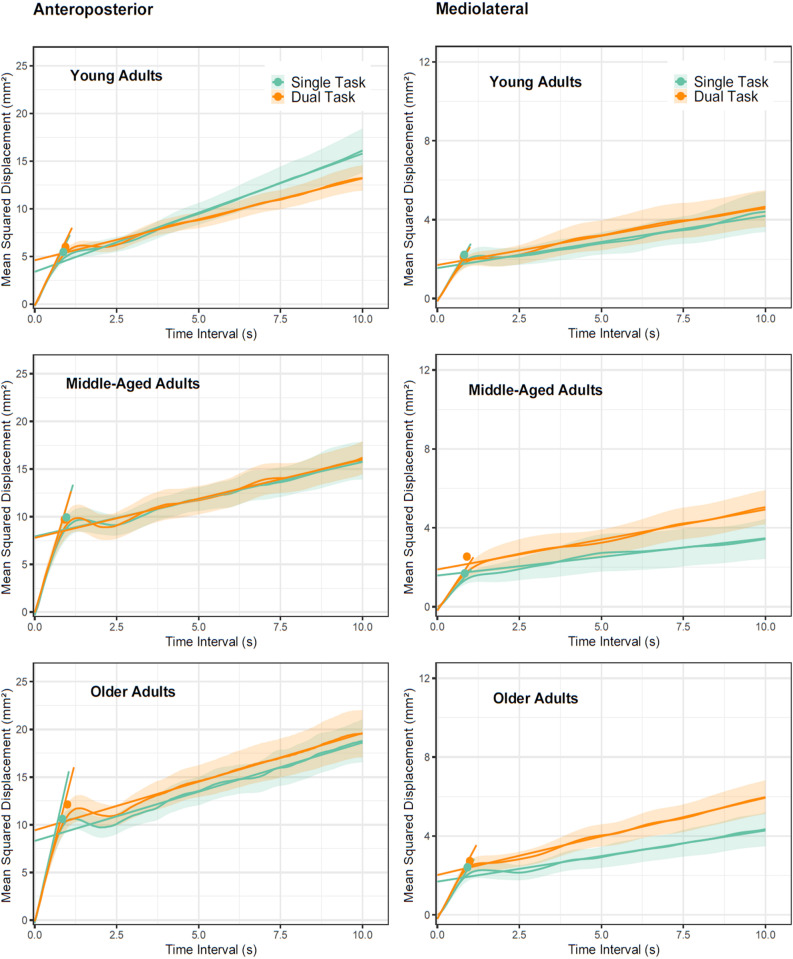
Stabilogram diffusion plots averaged across subjects within age groups for AP (left) and ML (right) COP sway. Fit lines represent estimates for short- and long-term diffusion coefficients in side-by-side quiet stance. Symbols denote the critical point. Error envelope = ± 1SE. Note that y-axes scales differ between AP and ML panels.

We used the parameters derived from our SDA approach to analyze the efficiency of postural control component processes and how they were affected by multitasking and cognitive control manipulations. Stabilogram diffusion functions for AP and ML displacement are shown in Fig 6. Slopes of displacements in approximately 0–1 sec ranges correspond to short-term diffusion. Slopes at larger delays correspond to long-term diffusion with shallower slopes indicating more efficient error corrections for displacements. Dots in each function indicate critical delays (y-axis) ad critical displacement (x-axis) corresponding to SDA estimates when error correction processes start dominating unsupervised short-term diffusion. Means and SD’s for SDA parameters are provided in Table 2. We analyzed AP and ML-sway separately because in our earlier study [45] ML-sway turned out to be more sensitive to age- and multitasking-related effects. As we detail below, this expectation was clearly supported in the data. Different from the predictions of the cognitive control account of multitasking, but in line with our findings for ellipse area, there was no evidence that the manipulation of cognitive control demands in the listening task (same vs. switching speakers) affected multitasking costs in postural control. For this reason, we maintained the factor in LMM’s but averaged the two dual-task conditions in Fig 6 and Table 2.

**Table 2:** Means and standard deviations of all postural control parameters for single and dual task conditions in all age groups. Note that these values do not depict the transformed measures used for statistical analysis.

		Young Adults		Middle-aged Adults		Older Adults	
**Measure**	**Condition**	**Mean**	**SD**	**Mean**	**SD**	**Mean**	**SD**
Ellipse area (mm²)	Single Task	30.74	21.62	27.08	24.89	38.22	26.23
	Dual Task	29.86	25.22	34.67	27.25	46.63	28.40
AP short-term diffusion coefficient (mm² s^-1^)	Single Task	3.23	1.51	5.25	3.01	7.12	3.13
	Dual Task	3.37	1.44	5.17	3.12	6.18	4.45
AP long-term diffusion coefficient (mm² s^-1^)	Single Task	0.49	0.22	0.29	0.16	0.42	0.37
	Dual Task	0.33	0.40	0.32	0.27	0.41	0.30
AP critical time interval (s)	Single Task	0.84	0.23	0.94	0.25	0.81	0.30
	Dual Task	0.90	0.31	0.89	0.29	0.96	0.23
AP critical mean squared displacement (mm²)	Single Task	5.07	2.04	9.17	6.42	10.10	5.86
	Dual Task	5.65	2.85	9.07	5.90	11.38	4.39
ML short-term diffusion coefficient (mm² s^-1^)	Single Task	1.33	0.79	0.89	0.79	1.25	0.95
	Dual Task	1.24	0.98	1.08	0.68	1.48	0.81
ML long-term diffusion coefficient (mm² s^-1^)	Single Task	0.12	0.10	0.08	0.08	0.12	0.11
	Dual Task	0.12	0.14	0.13	0.12	0.16	0.12
ML critical time interval (s)	Single Task	0.80	0.27	0.82	0.33	0.91	0.34
	Dual Task	0.77	0.26	0.86	0.30	0.97	0.31
ML critical mean squared displacement (mm²)	Single Task	2.07	1.75	1.51	2.48	2.17	1.65
	Dual Task	1.92	2.09	2.03	1.72	2.51	1.61

SD, standard deviation; AP, anteroposterior; ML, mediolateral.

Short-term diffusion in AP-sway showed the expected age effects with young adults showing less diffusion (z=3.664, p<.001) and critical displacement than middle-aged adults (z=3.507, p<.001). Likewise, older adults showed higher critical displacements than middle-aged adults (z=2.232, p=.029). In line with our earlier findings, older adults showed higher short-term diffusion compared with middle-aged adults, however, these effects did not reach significance (z=1.695, p=.095).

Nested effects of age indicated increased short-term diffusion and critical displacement with increasing age in middle-aged (z=2.562, p=.0128; z=3.225, p=.002) and older adults (z=2.844, p=.006; z=2.444, p=.017). A nested age by context effect revealed that in young adults, increasing age increased critical displacement in dual-task contexts (z=2.509, p= 0.015). Multitasking caused increased AP critical time intervals (z=2.377, p=.021) an effect that was higher in older compared with middle-aged participants (z=2.786, p=.007). Post-hoc tests showed that older adults were the only group reliably increasing critical AP delays under concurrent task demands (t(18)= 3.581, p=.002). A context by young vs middle-aged contrast pointed to increased long-term diffusion and decreased mean squared displacement under dual-task contexts in middle-aged adults (z=2.661, p=.008; z=2.063 p=.043). However, post-hoc tests did not corroborate this effect (t’s< 1.924, p’s>0.069). In sum, our SDA analysis of AP-sway replicated age effects found in earlier studies. Multitasking effects were limited to an increase in the critical time intervals in older adults.

A different picture emerged from the analysis of ML-sway. In line with our predictions, older adults showed more short-term diffusion (z=2.160, p=.035) and higher critical displacement (z=2.370, p=.021) than middle-aged adults. While middle-aged adults showed higher, though insignificant (z=1.602, p=0.114) short-term diffusion than young adults, other SDA parameters showed comparable levels between these age groups (z<0.772, p>.443).

We obtained a significant context effect for ML long-term diffusion (z=2.628, p=.011) which was qualified by a crossover interaction revealing that middle-aged adults showed pronounced multitasking-related increases in long-term slopes compared with young adults (z=2.296, p= 0.0253). Post-hoc tests revealed context-related increases in ML long-term diffusion for middle-aged (t(22)= 3.108, p=.005) but not young adults (t(20)=0.375, p=.712). Another interaction between context and age group (young vs. middle-aged) pointed to increased critical ML displacement during multitasking in middle-aged compared with younger adults (z=2.149, p=.036). Post-hoc tests established similar single- and dual-task performances in young adults (t(20)=0.926, p=.365) but failed to corroborate the interaction for middle-aged adults by a slight margin (t(22)=2.034, p=.054). No reliable interaction effects emerged when comparing critical ML displacement in older with middle-aged adults (z=0.467, p=.641), suggesting comparable multitasking-related performance decrements in both age groups. This was further corroborated by a robust single-dual task difference in older adults (t(18)=2.221, p=.039).

As mentioned above, control demands in the listening task did not affect context-related effects or related age-group differences. The only effect supporting our predictions was a nested age by cognitive control manipulation indicating higher critical ML displacement with increasing age in older adults (z=2.051, p=.041), which, however, was without consequence for multitasking. Somewhat unexpectedly we found lower AP short-term diffusion (z=2.924, p=.004) and AP critical displacement (z=3.034 p=.003) in the switching compared with the quiet stance condition. We obtained an interaction between the young vs middle-aged contrast and the cognitive control manipulation in AP critical time interval reflecting opposing trends in the two groups (z=2.028, p=.043). However, these differences could not be substantiated in post-hoc tests (t’s<1.905, p’s>0.070) suggesting the interaction was spurious.

Corroborating the results of smaller ellipse areas for female compared with male participants, we obtained several significant sex differences in SDA parameters, none of which affected the results reported above. Women had lower AP short-term diffusion than men (z=3.950, p<.001), a difference which was larger in middle-aged compared with young (z=2.176, p=.033) and older adults (z=2.179, p=.033). As a result, critical displacement was also lower in female compared with male participants (z=3.111, p=.003). Female participants had better error correction for AP sway as indicated by lower AP long-term diffusion (z=2.740, p=.008) and this difference was larger in single- than in dual-task contexts (z=2.515, p=.0121). ML-sway showed the same female advantage in short-term diffusion (z=2.368, p=.021) that we saw in AP-sway, however they did not differ between age groups. Critical time intervals and critical displacement for ML-sway were also higher in male participants (z=2.215, p=.030; z=3.138, p=.003). For ML-error correction (long-term diffusion) a sex by context interaction (z=2.685, p=.009) revealed a slight female advantage in single- but also larger costs for women in dual-task conditions. Lastly, we found sex differences for critical displacements to be larger in the same compared with switching speaker conditions (z=2.147, p=.032).

### Combined costs of multitasking

**Fig 7:**
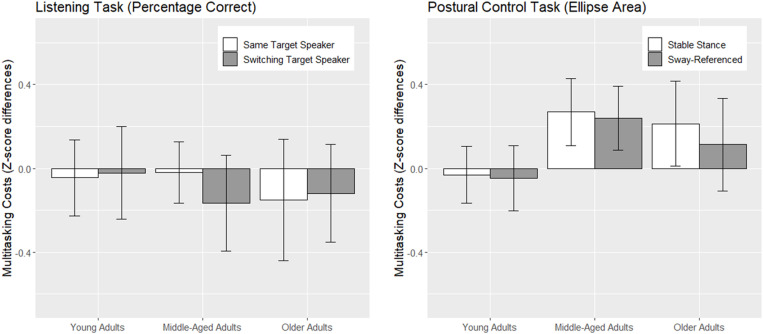
Multitasking costs based on z-transformed performance measures for each experimental condition.

To evaluate differences between age groups regarding overall multitasking costs and differences between task modalities, we used z-transformed performance measures. To this end we determined z-scores for each task and calculated z-score differences per subject by subtracting the mean dual-task performance in each task combination from the mean single-task performance. For postural control, we focus on the CoP ellipse area as this parameter showed sensitivity to concurrent task interference and applied to stable stance and sway-referenced conditions (Fig 7). In line with the findings reported before, we found higher multitasking costs for postural control compared with the listening task (z=4.452, p<.001). The young vs middle-aged contrast showed a main effect (z=2.208, p=.031) that was qualified by an interaction with modality (z=2.444, p=.017) reflecting the fact that middle-adults higher multitasking costs originated solely in the postural control task. Costs at this level were similar for middle-aged and older adults. Difficulty of concurrent tasks (stable vs sway-referencing; same vs switching target speakers) did not contribute to the model fit (S1 Appendix). We maintained the posture-task difficulty distinction in Fig 7 to illustrate that we found no evidence for prioritization in any of the age groups. Overall, female participants showed higher multitasking costs compared with male participants (z=2.332, p=.023). This finding corresponds to our results from SDA parameters which indicated female performance advantages, but also pointed to higher sensitivities for multitasking interference in women’s postural control.

## Discussion

We investigated adult-age changes in multitasking using a combination of listening and postural control tasks. Our paradigm simulated ecologically valid contexts like cocktail-party conversations and taxed abilities known to be susceptible to early age-related declines. Our main research questions targeted possible age-related declines in multitasking as early as middle-adulthood and the explanatory scope of cognitive compensation and cognitive control accounts of multitasking. To this end we manipulated cognitive control demands of the listening task and the difficulty of the postural control task. The latter manipulation was designed to elicit age-differential prioritization strategies.

We controlled for baseline differences in peripheral hearing abilities and we adjusted the working memory load to the individual’s single-task capacities. As a result of the latter adjustment older adults worked with shorter word lists than middle-aged adults. The adjustment was successful in avoiding floor and ceiling effects in all groups. However, while young and middle-aged adults showed similar levels of percentage correct recognition in single-task (same target speaker condition), older adults performed weaker than the other groups despite their lower task difficulty levels.

Manipulations of task difficulty in both postural control and listening tasks proved effective. As expected, listening task performance declined in all groups when participants needed to switch target speakers. In the postural control task, ellipse areas increased when the reliability of proprioceptive information was reduced (sway-referencing condition). Both difficulty manipulations had similar effects across age groups.

Performance on the listening task was unaffected by concurrent postural control demands. All three age groups maintained single-task levels of listening performances even when the postural control task was made more difficult. In contrast, concurrent listening caused substantial decrements in postural control in middle-aged and older but not in young adults. Summary statistics (ellipse area) indicated that middle-aged participants showed similar levels of postural control in single-task conditions as young adults did, however, concurrent listening reduced middle-aged adults’ stability whereas young adults remained basically unaffected. Older adults showed the poorest overall balance performance of all groups and their dual-task decrements were of similar magnitude as in middle-aged adults. Stabilogram diffusion analysis replicated our findings from earlier work [45] demonstrating that multitasking affects parameters reflecting timing and quality of postural adjustments (critical time interval and long-term diffusion). Like in the earlier study, ML long-term diffusion revealed an age-differential pattern sensitive to multitasking interference thereby substantiating the findings from the ellipse area.

Different from our expectations, increasing the cognitive control demands in the listening task (i.e., switching speaker condition) did not increase multitasking decrements. In contrast, the only significant effect of the cognitive control manipulation on concurrent task performance was a decrease in AP short-term diffusion and critical displacement. Related findings have sometimes been interpreted according to the action constraint hypothesis [72], which suggests that demanding dual-task conditions divert focus away from postural control, thereby facilitating more effective automatic regulation of our upright stability. However, this explanation does not align with our observation of poorer postural control performance under dual-task conditions. The postural control difficulty manipulation did not produce the anticipated results concerning multitasking either, as it failed to trigger a shift in task prioritization. All groups, including older adults, showed perfect preservation of listening performance under multitasking conditions. A possible explanation is that the sway-referenced condition was not jeopardizing stability to the point where protective postural control strategies were elicited. Previous studies using similar manipulations differed regarding observing prioritization [61,62] or not [73,74].

Taken together our results provide no evidence in support of the cognitive control account of multitasking. Obviously, such a null finding does not rule out cognitive control accounts altogether. After all, our listening task had a cognitive control component (inhibition) even in its simplest version. The absence of the predicted age group by switching interaction already in the single-task condition suggests that the relative increase in cognitive control demands could be compensated by older adults who might have benefitted from the combination of shorter stimulus lists and longer reaction times.

Instead of a cognitive control account, we argue that our results are best explained by a version of the cognitive compensation account. Specifically, we consider compensatory mechanisms as underlying middle-aged adults’ maintenance of near-young adult levels of stability in single-task contexts. The breakdown of these very same mechanisms under dual-task conditions subsequently leads to pronounced costs in this group.

Naturally, the question arises why compensation was more prominent in middle-aged than the other age groups? Compensation accounts have a long tradition in aging research and neuropsychology [75–77]. According to related models (e.g., CRUNCH, scaffolding) compensation mechanisms are at the disposal of all age groups; however, utilization of related mechanisms and strategies does not come for free but at a (processing) cost [76,78]. Whether a certain individual can utilize compensatory mechanisms thus depends on task challenges and individual differences in available processing resources. As a result, compensation can be effective only up to a certain threshold of task demands beyond which performance inevitably deteriorates.

Dual-task decrements in middle-aged adults were at least as pronounced as those observed in older adults, if not greater. In our view this indicates that while middle-aged adults were more proficient in employing compensation strategies, they were also more vulnerable to their disruption. We argue that under multitasking conditions concurrent task demands surpass this threshold even for middle-aged adults leading to significantly greater dual-task decrements than in younger counterparts.

The accumulating evidence for developmental changes in postural control and related changes in multitasking emphasizes the value of using non-linear methods like SDA over traditional summary statistics like path length or sway area. A limitation of our study in this respect is that SDA is not suitable for analyzing center-of-pressure trajectories obtained from a sway-referenced platform. Future studies should incorporate difficulty manipulations that allow for a distinction between the various control processes involved in postural control. Additionally, despite our efforts to equate the difficulty of the listening task under single-task conditions, older adults still performed worse, which may have influenced the magnitude of dual-task-related decrements in this age group.

In conclusion, our study demonstrated that real-life multitasking settings involving sensorimotor processing with an early onset of age-related decline induce elevated costs in middle-aged adults already. This decline in multitasking arises from a breakdown of compensatory processes rather than a direct change in multitasking abilities. Our findings offer insights into how middle-aged adults maintain high functioning despite age-related changes, highlighting the potential as well as the limitations of their compensatory strategies.

## Supporting information

S1 Appendix Variability in listening task strategy. (DOCX)

S2 Appendix Model selection. (DOCX)

S3 Appendix Tables of numerical values of results. (DOCX)

## References

[1] Mattys SL, Davis MH, Bradlow AR, Scott SK. Speech recognition in adverse conditions: a review. Lang Cogn Process. 2012;27(7–8):953–78. doi: 10.1080/01690965.2012.705006

[2] Pichora-Fuller MK, Singh G. Effects of age on auditory and cognitive processing: implications for hearing aid fitting and audiologic rehabilitation. Trends Amplif. 2006;10(1):29–59. doi: 10.1177/108471380601000103 16528429 PMC4111543

[3] Kiessling J, Pichora-Fuller MK, Gatehouse S, Stephens D, Arlinger S, Chisolm T, et al. Candidature for and delivery of audiological services: special needs of older people. Int J Audiol. 2003;42 Suppl 2:2S92–101. doi: 10.3109/14992020309074650 12918635

[4] Sussman ES. Auditory scene analysis: an attention perspective. J Speech Lang Hear Res. 2017;60(10):2989–3000. doi: 10.1044/2017_JSLHR-H-17-0041 29049599 PMC5945068

[5] Boisgontier MP, Beets IAM, Duysens J, Nieuwboer A, Krampe RT, Swinnen SP. Age-related differences in attentional cost associated with postural dual tasks: increased recruitment of generic cognitive resources in older adults. Neurosci Biobehav Rev. 2013;37(8):1824–37. doi: 10.1016/j.neubiorev.2013.07.014 23911924

[6] Helfer KS, Freyman RL, van Emmerik R, Banks J. Postural control while listening in younger and middle-aged adults. Ear Hear. 2020;41(5):1383–96. doi: 10.1097/AUD.0000000000000861 32149925 PMC8034820

[7] Helfer KS, van Emmerik R, Banks JJ, Freyman RL. Early aging and postural control while listening and responding. J Acoust Soc Am. 2020;148(5):3117. doi: 10.1121/10.0002485 33261409 PMC7690971

[8] Lindenberger U, Marsiske M, Baltes PB. Memorizing while walking: increase in dual-task costs from young adulthood to old age. Psychol Aging. 2000;15(3):417–36. doi: 10.1037//0882-7974.15.3.417 11014706

[9] Naaman T, Hayek R, Gutman I, Springer S. Young, but not in the dark-the influence of reduced lighting on gait stability in middle-aged adults. PLoS One. 2023;18(5):e0280535. doi: 10.1371/journal.pone.0280535 37200289 PMC10194872

[10] Prado JM, Stoffregen TA, Duarte M. Postural sway during dual tasks in young and elderly adults. Gerontology. 2007;53(5):274–81. doi: 10.1159/000102938 17510558

[11] Sommers MS. Listening comprehension in middle-aged adults. Am J Audiol. 2015;24(2):88–90. doi: 10.1044/2015_AJA-14-0060 25768392 PMC4610268

[12] Schaie KW. The course of adult intellectual development. Am Psychol. 1994;49(4):304–13. doi: 10.1037//0003-066x.49.4.304 8203802

[13] Metter EJ, Conwit R, Tobin J, Fozard JL. Age-associated loss of power and strength in the upper extremities in women and men. J Gerontol A Biol Sci Med Sci. 1997;52(5):B267–76. doi: 10.1093/gerona/52a.5.b267 9310077

[14] Arvin B, Prepageran N, Raman R. “High frequency presbycusis”-is there an earlier onset? Indian J Otolaryngol Head Neck Surg. 2013;65(Suppl 3):480–4. doi: 10.1007/s12070-011-0356-x 24427701 PMC3889367

[15] Vercammen C, Goossens T, Wouters J, van Wieringen A. Digit triplet test hearing screening with broadband and low-pass filtered noise in a middle-aged population. Ear Hear. 2018;39(4):825–8. doi: 10.1097/AUD.0000000000000524 29189521

[16] Van Wilderode M, Van Humbeeck N, Krampe R, van Wieringen A. Speech-identification during standing as a multitasking challenge for young, middle-aged and older adults. Trends Hear. 2024;28:23312165241260621. doi: 10.1177/23312165241260621 39053897 PMC11282555

[17] Kahneman D. Attention and effort. 1973.

[18] Navon D. Resources—a theoretical soup stone? Psychol Rev. 1984;91(2):216–34. doi: 10.1037/0033-295x.91.2.216

[19] Dux PE, Ivanoff J, Asplund CL, Marois R. Isolation of a central bottleneck of information processing with time-resolved FMRI. Neuron. 2006;52(6):1109–20. doi: 10.1016/j.neuron.2006.11.009 17178412 PMC2527865

[20] Deprez S, Vandenbulcke M, Peeters R, Emsell L, Amant F, Sunaert S. The functional neuroanatomy of multitasking: combining dual tasking with a short term memory task. Neuropsychologia. 2013;51(11):2251–60. doi: 10.1016/j.neuropsychologia.2013.07.024 23938320

[21] Meijer A-M, Krampe RT. Movement timing and cognitive control: adult-age differences in multi-tasking. Psychol Res. 2018;82(1):203–14. doi: 10.1007/s00426-017-0876-4 28624969

[22] Allen PA, Smith AF, Vires-Collins H, Sperry S. The psychological refractory period: evidence for age differences in attentional time-sharing. Psychol Aging. 1998;13(2):218–29. doi: 10.1037//0882-7974.13.2.218 9640583

[23] Glass JM, Schumacher EH, Lauber EJ, Zurbriggen EL, Gmeindl L, Kieras DE, et al. Aging and the psychological refractory period: task-coordination strategies in young and old adults. Psychol Aging. 2000;15(4):571–95. doi: 10.1037//0882-7974.15.4.571 11144318

[24] Wasylyshyn C, Verhaeghen P, Sliwinski MJ. Aging and task switching: a meta-analysis. Psychol Aging. 2011;26(1):15–20. doi: 10.1037/a0020912 21261411 PMC4374429

[25] Christ SE, White DA, Mandernach T, Keys BA. Inhibitory control across the life span. Dev Neuropsychol. 2001;20(3):653–69. doi: 10.1207/S15326942DN2003_7 12002099

[26] Ruffieux J, Keller M, Lauber B, Taube W. Changes in standing and walking performance under dual-task conditions across the lifespan. Sports Med. 2015;45(12):1739–58. doi: 10.1007/s40279-015-0369-9 26253187 PMC4656695

[27] van Wieringen A, Van Wilderode M, Van Humbeeck N, Krampe R. Coupling of sensorimotor and cognitive functions in middle- and late adulthood. Front Neurosci. 2022;16:1049639. doi: 10.3389/fnins.2022.1049639 36532286 PMC9752872

[28] Verhaeghen P, Cerella J. Aging, executive control, and attention: a review of meta-analyses. Neurosci Biobehav Rev. 2002;26(7):849–57. doi: 10.1016/s0149-7634(02)00071-4 12470697

[29] Uchida Y, Sugiura S, Nishita Y, Saji N, Sone M, Ueda H. Age-related hearing loss and cognitive decline - the potential mechanisms linking the two. Auris Nasus Larynx. 2019;46(1):1–9. doi: 10.1016/j.anl.2018.08.010 30177417

[30] Li KZH, Lindenberger U. Relations between aging sensory/sensorimotor and cognitive functions. Neurosci Biobehav Rev. 2002;26(7):777–83. doi: 10.1016/s0149-7634(02)00073-8 12470689

[31] Zettel-Watson L, Suen M, Wehbe L, Rutledge DN, Cherry BJ. Aging well: Processing speed inhibition and working memory related to balance and aerobic endurance. Geriatr Gerontol Int. 2017;17(1):108–15. 26694752 10.1111/ggi.12682

[32] Holtzer R, Friedman R, Lipton RB, Katz M, Xue X, Verghese J. The relationship between specific cognitive functions and falls in aging. Neuropsychology. 2007;21(5):540–8. doi: 10.1037/0894-4105.21.5.540 17784802 PMC3476056

[33] Albers MW, Gilmore GC, Kaye J, Murphy C, Wingfield A, Bennett DA, et al. At the interface of sensory and motor dysfunctions and Alzheimer’s disease. Alzheimers Dement. 2015;11(1):70–98. doi: 10.1016/j.jalz.2014.04.514 25022540 PMC4287457

[34] Fulton SE, Lister JJ, Bush ALH, Edwards JD, Andel R. Mechanisms of the hearing-cognition relationship. Semin Hear. 2015;36(3):140–9. doi: 10.1055/s-0035-1555117 27516714 PMC4906307

[35] Mahoney J, Verghese J. Does cognitive impairment influence visual-somatosensory integration and mobility in older adults? J Gerontol: Series A, Biol Sci Med Sci. 2020;75(3):581–8. 10.1093/gerona/glz117 PMC7328197 31111868

[36] Campos JL, Marusic U, Mahoney JR. Editorial: the intersection of cognitive, motor, and sensory processing in aging: links to functional outcomes, Volume I. Fronti Aging Neurosci. 2022;14. 10.3389/fnagi.2022.1009532 PMC9470213 36110431

[37] Livingston G, Sommerlad A, Orgeta V, Costafreda SG, Huntley J, Ames D, et al. Dementia prevention, intervention, and care. Lancet. 2017;390(10113):2673–734. doi: 10.1016/S0140-6736(17)31363-6 28735855

[38] Verghese J, Ayers E, Barzilai N, Bennett DA, Buchman AS, Holtzer R, et al. Motoric cognitive risk syndrome: multicenter incidence study. Neurology. 2014;83(24):2278–84. doi: 10.1212/WNL.0000000000001084 25361778 PMC4277675

[39] Heuninckx S, Wenderoth N, Debaere F, Peeters R, Swinnen S. Neural basis of aging: the penetration of cognition into action control. J Neurosci. 2005;25(29):6787–96. 16033888 10.1523/JNEUROSCI.1263-05.2005 PMC6725362

[40] Heuninckx S, Wenderoth N, Swinnen S. Systems neuroplasticity in the aging brain: recruiting additional neural resources for successful motor performance in elderly persons. J Neurosci. 2008;28(1):91–9. 18171926 10.1523/JNEUROSCI.3300-07.2008 PMC6671150

[41] Heuninckx S, Wenderoth N, Swinnen SP. Age-related reduction in the differential pathways involved in internal and external movement generation. Neurobiol Aging. 2010;31(2):301–14. doi: 10.1016/j.neurobiolaging.2008.03.021 18472185

[42] Goble DJ, Coxon JP, Van Impe A, De Vos J, Wenderoth N, Swinnen SP. The neural control of bimanual movements in the elderly: brain regions exhibiting age-related increases in activity, frequency-induced neural modulation, and task-specific compensatory recruitment. Hum Brain Mapp. 2010;31(8):1281–95. doi: 10.1002/hbm.20943 20082331 PMC6871108

[43] St George RJ, Jayakody O, Healey R, Breslin M, Hinder MR, Callisaya ML. Cognitive inhibition tasks interfere with dual-task walking and increase prefrontal cortical activity more than working memory tasks in young and older adults. Gait Posture. 2022;95:186–91. doi: 10.1016/j.gaitpost.2022.04.021 35525151

[44] Anderson M, Bucks RS, Bayliss DM, Della Sala S. Effect of age on dual-task performance in children and adults. Mem Cognit. 2011;39(7):1241–52. doi: 10.3758/s13421-011-0099-7 21538179

[45] Van Humbeeck N, Van Wilderode M, Kliegl R, van Wieringen A, Krampe RT. Multitasking across the lifespan in different task contexts. Sci Rep. 2024;14(1):11817. doi: 10.1038/s41598-024-61859-w 38783047 PMC11116417

[46] Li KZ, Lindenberger U, Freund AM, Baltes PB. Walking while memorizing: age-related differences in compensatory behavior. Psychol Sci. 2001;12(3):230–7. doi: 10.1111/1467-9280.00341 11437306

[47] Fernandez-Cervantes E, Montesinos L, Gonzalez-Nucamendi A, Pecchia L. Recurrence quantification analysis of center of pressure trajectories for balance and fall-risk assessment in young and older adults. IEEE Trans Neural Syst Rehabil Eng. 2023. doi: 10.1109/TNSRE.2023.3236454 37018724

[48] Van Humbeeck N, Kliegl R, Krampe RT. Lifespan changes in postural control. Sci Rep. 2023;13(1):541. doi: 10.1038/s41598-022-26934-0 36631521 PMC9834247

[49] Mengarelli A, Tigrini A, Verdini F, Rabini R, Fioretti S. Multiscale fuzzy entropy analysis of balance: Evidences of scale-dependent dynamics on diabetic patients with and without neuropathy. IEEE Trans Neural Syst Rehabil Eng. 2023. 10.1109/TNSRE.2023.3248322 37027606

[50] Lacour M, Bernard-Demanze L, Dumitrescu M. Posture control, aging, and attention resources: models and posture-analysis methods. Neurophysiol Clin. 2008;38(6):411–21. doi: 10.1016/j.neucli.2008.09.005 19026961

[51] Gilfriche P, Deschodt-Arsac V, Blons E, Arsac LM. Frequency-specific fractal analysis of postural control accounts for control strategies. Front Physiol. 2018;9. 29643816 10.3389/fphys.2018.00293 PMC5883185

[52] Collins JJ, De Luca CJ. Open-loop and closed-loop control of posture: a random-walk analysis of center-of-pressure trajectories. Exp Brain Res. 1993;95(2):308–18. doi: 10.1007/BF00229788 8224055

[53] Collins JJ, De Luca CJ, Burrows A, Lipsitz LA. Age-related changes in open-loop and closed-loop postural control mechanisms. Exp Brain Res. 1995;104(3):480–92. doi: 10.1007/BF00231982 7589299

[54] Collins JJ, De Luca CJ. The effects of visual input on open-loop and closed-loop postural control mechanisms. Exp Brain Res. 1995;103(1):151–63. doi: 10.1007/BF00241972 7615030

[55] Winter D, Prince F, Frank J, Powell C, Zabjek K. Unified theory regarding A/P and M/L balance in quiet stance. J Neurophysiol. 1996;75(6):2334–43. 8793746 10.1152/jn.1996.75.6.2334

[56] Stel VS, Smit JH, Pluijm SMF, Lips P. Balance and mobility performance as treatable risk factors for recurrent falling in older persons. J Clin Epidemiol. 2003;56(7):659–68. doi: 10.1016/s0895-4356(03)00082-9 12921935

[57] Slobounov S, Hallett M, Cao C, Newell K. Modulation of cortical activity as a result of voluntary postural sway direction: an EEG study. Neurosci Lett. 2008;442(3):309–13. 18639613 10.1016/j.neulet.2008.07.021 PMC2546523

[58] Maki BE, Holliday PJ, Topper AK. A prospective study of postural balance and risk of falling in an ambulatory and independent elderly population. J Gerontol. 1994;49(2):M72–84. doi: 10.1093/geronj/49.2.m72 8126355

[59] Huff MJ, Balota DA, Minear M, Aschenbrenner AJ, Duchek JM. Dissociative global and local task-switching costs across younger adults, middle-aged adults, older adults, and very mild Alzheimer’s disease individuals. Psychol Aging. 2015;30(4):727–39. doi: 10.1037/pag0000057 26652720 PMC4681312

[60] Kray J, Eber J, Karbach J. Verbal self-instructions in task switching: a compensatory tool for action-control deficits in childhood and old age? Dev Sci. 2008;11(2):223–36. doi: 10.1111/j.1467-7687.2008.00673.x 18333979

[61] Doumas M, Smolders C, Krampe RT. Task prioritization in aging: effects of sensory information on concurrent posture and memory performance. Exp Brain Res. 2008;187(2):275–81. doi: 10.1007/s00221-008-1302-3 18273609

[62] Rapp MA, Krampe RT, Baltes PB. Adaptive task prioritization in aging: selective resource allocation to postural control is preserved in Alzheimer disease. Am J Geriatr Psychiatry. 2006;14(1):52–61. doi: 10.1097/01.JGP.0000192490.43179.e7 16407582

[63] Doumas M, Krampe RT. Ecological relevance determines task priority in older adults’ multitasking. J Gerontol B Psychol Sci Soc Sci. 2015;70(3):377–85. doi: 10.1093/geronb/gbt105 24149518

[64] Ziso B, Larner AJ. Codex (cognitive disorders examination) decision tree modified for the detection of dementia and MCI. Diagnostics (Basel). 2019;9(2). 10.3390/diagnostics9020058 PMC6628135 31159432

[65] Hemmelmann C, Brose S, Vens M, Hebebrand J, Ziegler A. Percentiles of body mass index of 18-80-year-old German adults based on data from the second national nutrition survey. Dtsch Med Wochenschr. 2010;135(17):848–52. 20408102 10.1055/s-0030-1253666

[66] Francart T, Hofmann M, Vanthornhout J, Van Deun L, van Wieringen A, Wouters J. APEX/SPIN: a free test platform to measure speech intelligibility. Int J Audiol. 2016;56:1–7. 27796135 10.1080/14992027.2016.1247215

[67] Brysbaert M, Stevens M, Mandera P, Keuleers E. How many words do we know? practical estimates of vocabulary size dependent on word definition, the degree of language input and the participant’s age. Front Psychol. 2016;7:1116. doi: 10.3389/fpsyg.2016.01116 27524974 PMC4965448

[68] Koltermann JJ, Gerber M, Beck H, Beck M. Validation of various filters and sampling parameters for a COP analysis. Technologies. 2018;6(2):56. doi: 10.3390/technologies6020056

[69] Oliveira LF, Simpson DM, Nadal J. Calculation of area of stabilometric signals using principal component analysis. Physiol Meas. 1996;17(4):305–12. doi: 10.1088/0967-3334/17/4/008 8953629

[70] Bates D, Kliegl R, Vasishth S, Baayen H. Parsimonious mixed models. arXiv. 2015;1506.

[71] Schad DJ, Vasishth S, Hohenstein S, Kliegl R. How to capitalize on a priori contrasts in linear (mixed) models: a tutorial. J Mem Lang. 2020;110:104038. doi: 10.1016/j.jml.2019.104038

[72] Huxhold O, Li S-C, Schmiedek F, Lindenberger U. Dual-tasking postural control: aging and the effects of cognitive demand in conjunction with focus of attention. Brain Res Bull. 2006;69(3):294–305. doi: 10.1016/j.brainresbull.2006.01.002 16564425

[73] Smolders C, Doumas M, Krampe RT. Posture and cognition interfere in later adulthood even without concurrent response production. Hum Mov Sci. 2010;29(5):809–19. doi: 10.1016/j.humov.2009.07.009 19786311

[74] Shumway-Cook A, Woollacott M, Kerns KA, Baldwin M. The effects of two types of cognitive tasks on postural stability in older adults with and without a history of falls. J Gerontol A Biol Sci Med Sci. 1997;52(4):M232–40. doi: 10.1093/gerona/52a.4.m232 9224435

[75] Cabeza R, Grady CL, Nyberg L, McIntosh AR, Tulving E, Kapur S, et al. Age-related differences in neural activity during memory encoding and retrieval: a positron emission tomography study. J Neurosci. 1997;17(1):391–400. doi: 10.1523/JNEUROSCI.17-01-00391.1997 8987764 PMC6793692

[76] Reuter-Lorenz PA, Cappell KA. Neurocognitive aging and the compensation hypothesis. Curr Dir Psychol Sci. 2008;17(3):177–82. doi: 10.1111/j.1467-8721.2008.00570.x

[77] Grady C. The cognitive neuroscience of ageing. Nat Rev Neurosci. 2012;13(7):491–505. 22714020 10.1038/nrn3256 PMC3800175

[78] Park DC, Reuter-Lorenz P. The adaptive brain: aging and neurocognitive scaffolding. Annu Rev Psychol. 2009;60:173–96. doi: 10.1146/annurev.psych.59.103006.093656 19035823 PMC3359129

