# Supplementary material for: Concurrent listening impairs compensatory postural control mechanisms in middle and late adulthood
Source: PLoS One. 2025 Apr 30;20(4):e0321828. doi: 10.1371/journal.pone.0321828 (PMC12043164; doi:10.1371/journal.pone.0321828)
Supplement: S1 Appendix — (DOCX) [file pone.0321828.s001.docx]

# **S1 Appendix - Variability in listening task strategy**

Building on the analysis of the listening task in the main manuscript, we conducted additional analyses incorporating the factor of word category (target vs non-target) to explore the degree to which differences in response times and task strategies (i.e., identifying targets vs inhibiting nontargets) can further explain our results (Fig A). Apart from corroborating the findings of previous analysis, results revealed better performance on the target when compared with the non-target words (z=5.659, p<.001). This effect was qualified by an interaction implying that older adults were pronouncedly poorer at inhibiting non-target words when compared with middle-aged adults (i.e., larger discrepancy between percentages of target hits and non-target rejection; z=9.824, p<.001). In addition, a nested age-effect revealed that the ability to inhibit non-target words decreased with increasing age (z=7.498, p<.001). Lastly, an interaction between the word category and the difficulty manipulation indicated that these differences between hits and correct rejections was pronounced in the more difficult switching-speaker condition (z=6.074, p<.001). Analysis of the reaction times revealed shorter response times in dual when compared with single-task contexts (z=5.406, p<.001). An interaction revealed that this was pronounced in the switching-target speaker when compared with the same-target speaker condition (z=2.083, p=.037). Young adults responded faster than middle-aged adults (z=4.451, p<.001), and middle-aged adults responded faster than older adults (z=2.143, p=.036). Response times depended on the task difficulty, showing faster responses in the same target-speaker when compared with the switching-target speaker condition (z=9.916, p<.001). Responses were faster for target than on non-target words (z=10.005, p<.001), and this was quantified by an interaction showing that this effect was larger in the middle-aged when compared with young adults (z=2.448, p=.014). A nested age effect in older adults revealed that the difference between response times on target and non-target words became larger with increasing age (z=4.195, p<.001).


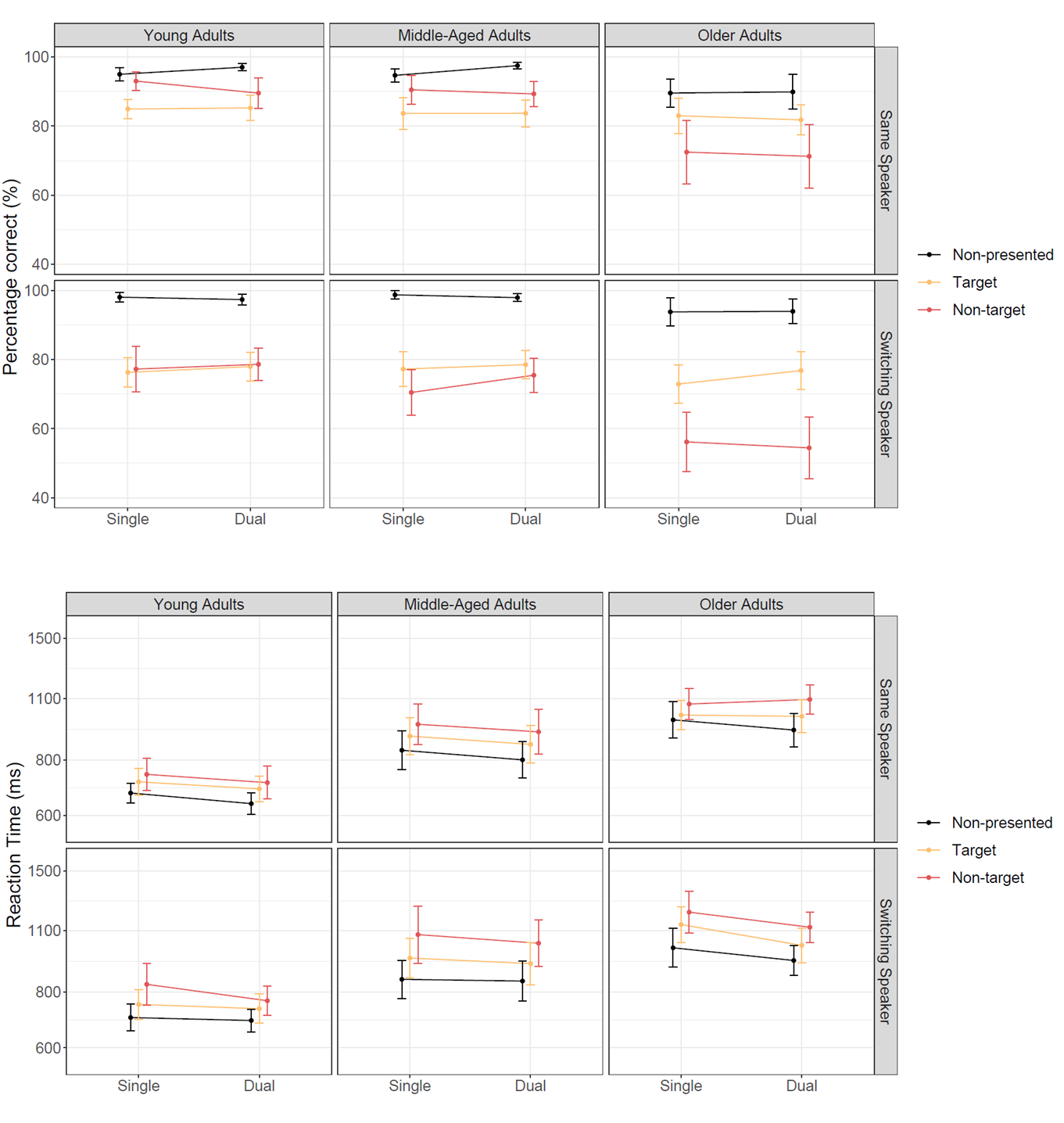


Fig A. Percentages correct and reaction times of non-presented, target and non-target words for each task context (Single and dual) under same and switching target speaker conditions. Error bars represent 95% between-group confidence intervals.
