# Supplementary material for: Concurrent listening impairs compensatory postural control mechanisms in middle and late adulthood
Source: PLoS One. 2025 Apr 30;20(4):e0321828. doi: 10.1371/journal.pone.0321828 (PMC12043164; doi:10.1371/journal.pone.0321828)
Supplement: S2 Appendix — (DOCX) [file pone.0321828.s002.docx]

# **S2 Appendix – Model selection**

For models involving postural control, nested effects of sex improved the model fit in all parameters (χ2(1)≥ 5.465, p<0.05) except from the AP critical time interval (χ2(1) = 2.932, p=0.087). Sex by context interactions contributed to the model fit in ML long-term diffusion (χ2(2)=10.521, p<0.001) and ML critical mean squared displacement (χ2(2)=6.354, p=0.042). Nested age contributed to the model fit in AP short-term diffusion (χ2(3)=13.176, p=0.004). Nested age by context interactions contributed significantly in the AP and ML critical mean squared displacement (χ2(6)= 15.351, p= 0.018; χ2(6) =13.926, p=0.030). Nested age by platform interactions revealed robust contributions in the ellipse area (χ2(3)= 10.554, p= 0.014). Model selection of the listening task indicated significant contributions of nested age (χ2(3)=20.04, p<0.001) but not sex (χ2(1)= 1.253, p=0.164). The LMM involving the combined multitasking costs did not show reliable contributions of the secondary task (χ2(1)= 1.178, p= 0.278) and nested age (χ2(3)=1.188, p=0.756). Sex by measure and sex by age group interactions contributed significantly (χ2(2)>11.200, p<0.005). Expanding the random effect structure by allowing subject by difficulty interactions improved the ellipse area model (χ2(1)= 89.392 , p<0.001). Allowing an expanded random effect structure including the task modality in the combined costs analysis contributed significantly to the model fit (χ2(1)= 4.218, p=0.040). Expanding the listening task model by allowing a subject by difficulty interaction improved the model (χ2(1)= 6.005, p=0.014). For the SDA models, expanding the random effect structure by allowing subject by context interactions improved all models (χ2(1)>4.609, p<0.032) except from the AP short-term diffusion, AP long-term diffusion, and ML critical time intervals (χ2(1)>1.892, p>0.169).
