# Supplementary material for: Concurrent listening impairs compensatory postural control mechanisms in middle and late adulthood
Source: PLoS One. 2025 Apr 30;20(4):e0321828. doi: 10.1371/journal.pone.0321828 (PMC12043164; doi:10.1371/journal.pone.0321828)
Supplement: S3 Appendix — (DOCX) [file pone.0321828.s003.docx]

# **S3 Appendix – Tables of numerical values of results**

|  |  |  |  |  |  |  |  |
| --- | --- | --- | --- | --- | --- | --- | --- |
| **Age group** | **Condition** | **Same speaker** | | | **Switching speaker** | | |
|  |  | **Mean** | **EB lower limit** | **EB upper limit** | **Mean** | **EB lower limit** | **EB upper limit** |
| Young Adults | Single Task | 89.48 | 87.61 | 91.35 | 83.33 | 80.59 | 86.07 |
|  | DT - Quiet | 90.93 | 87.77 | 94.09 | 82.60 | 79.19 | 86.00 |
|  | DT - Sway | 89.52 | 86.76 | 92.29 | 85.01 | 81.79 | 88.23 |
| Middle-aged Adults | Single Task | 88.47 | 85.81 | 91.13 | 81.15 | 77.46 | 84.84 |
|  | DT - Quiet | 90.19 | 87.67 | 92.70 | 84.02 | 80.74 | 87.30 |
|  | DT - Sway | 87.39 | 84.24 | 90.53 | 81.91 | 79.11 | 84.71 |
| Older Adults | Single Task | 82.55 | 77.85 | 87.25 | 75.77 | 72.01 | 79.52 |
|  | DT - Quiet | 84.96 | 79.94 | 89.99 | 78.15 | 73.28 | 83.02 |
|  | DT - Sway | 83.74 | 79.22 | 88.26 | 75.68 | 71.78 | 79.57 |
|  |  |  |  |  |  |  |  |

Table 1. Numerical values of results depicted in Fig 4. EB denotes the error bar.

|  |  |  |  |  |  |  |  |
| --- | --- | --- | --- | --- | --- | --- | --- |
| **Age group** | **Condition** | **Quiet Stance** | | | **Sway-Referenced** | | |
|  |  | **Mean** | **EB lower limit** | **EB upper limit** | **Mean** | **EB lower limit** | **EB upper limit** |
| Young Adults | Single Task | 30.74 | 23.53 | 40.16 | 79.87 | 61.93 | 103.00 |
|  | Dual Task | 29.86 | 23.38 | 38.15 | 76.42 | 57.02 | 102.42 |
| Middle-aged Adults | Single Task | 27.08 | 20.90 | 35.10 | 79.16 | 58.93 | 106.33 |
|  | Dual Task | 34.67 | 26.38 | 45.57 | 98.79 | 72.38 | 134.83 |
| Older Adults | Single Task | 38.22 | 29.20 | 50.02 | 118.30 | 88.94 | 157.35 |
|  | Dual Task | 46.63 | 37.65 | 57.75 | 131.68 | 107.31 | 161.58 |
|  |  |  |  |  |  |  |  |

Table 2. Numerical values of results depicted in Fig 5. EB denotes the error bar. Note that the means and standard deviations were calculated in log-space.

|  |  |  |  |  |  |
| --- | --- | --- | --- | --- | --- |
| **Age group** | **Condition** | **Mean** | **EB lower limit** | **EB upper limit** |  |
|  |  |  |  |  |  |
| Young Adults | Stable Stance | -0.03 | -0.17 | 0.10 |  |
|  | Sway-Referenced | -0.05 | -0.20 | 0.11 |  |
| Middle-aged Adults | Stable Stance | 0.27 | 0.11 | 0.43 |  |
|  | Sway-Referenced | 0.24 | 0.09 | 0.39 |  |
| Older Adults | Stable Stance | 0.21 | 0.01 | 0.42 |  |
|  | Sway-Referenced | 0.11 | -0.11 | 0.34 |  |
|  |  |  |  |  |  |

Table 3. Numerical values of postural control results depicted in Fig 7. EB denotes the error bar.

|  |  |  |  |  |  |
| --- | --- | --- | --- | --- | --- |
| **Age group** | **Condition** | **Mean** | **EB lower limit** | **EB upper limit** |  |
|  |  |  |  |  |  |
| Young Adults | Same Speaker | -0.05 | -0.23 | 0.14 |  |
|  | Switching Speaker | -0.02 | -0.24 | 0.20 |  |
| Middle-aged Adults | Same Speaker | -0.02 | -0.17 | 0.13 |  |
|  | Switching Speaker | -0.17 | -0.39 | 0.06 |  |
| Older Adults | Same Speaker | -0.15 | -0.44 | 0.14 |  |
|  | Switching Speaker | -0.12 | -0.35 | 0.11 |  |
|  |  |  |  |  |  |

Table 4. Numerical values of listening results depicted in Fig 7. EB denotes the error bar.
